# Approaches and genetic determinants in predicting response to neoadjuvant chemotherapy in locally advanced gastric cancer

**DOI:** 10.18632/oncotarget.12955

**Published:** 2016-10-27

**Authors:** Jichun Zhou, Jianguo Shen, Benjamin J. Seifer, Shaojie Jiang, Ji Wang, Hanchu Xiong, Lingmin Xie, Linbo Wang, Xinbing Sui

**Affiliations:** ^1^ Department of Surgical Oncology, Sir Run Run Shaw Hospital, Zhejiang University, Hangzhou, Zhejiang, China; ^2^ Biomedical Research Center and Key Laboratory of Biotherapy of Zhejiang Province, Hangzhou, Zhejiang, China; ^3^ Department of Obstetrics, Gynecology and Reproductive Sciences, Yale University School of Medicine, New Haven, CT, USA; ^4^ Department of Radiology, Sir Run Run Shaw Hospital, Zhejiang University, Hangzhou, Zhejiang, China; ^5^ Department of Medical Oncology, Sir Run Run Shaw Hospital, Zhejiang University, Hangzhou, Zhejiang, China

**Keywords:** neoadjuvant chemotherapy, gastric cancer, predictive biomarker, histopathological response, chemosensitivity

## Abstract

Gastric cancer remains a major health burden worldwide. There is near-universal agreement that neoadjuvant chemotherapy (NAC) is a preferred management for locally advanced gastric cancer (LAGC). However, the optimal approach for an individual patient is still not clear and remains controversial, which could be at least partly explained by the lack of predictive tools. The ability to predict chemosensitivity from NAC in routine clinical practice is difficult and is an area of intense investigation, especially in the Precision-Medicine Era. Available consistent evidence suggests that a favorable tumor histopathological response to NAC may be a useful positive prognostic marker in gastric cancer. Hence, it is reasonable to speculate that making the histopathological response from NAC predictable will dramatically facility the NAC and improve patients outcome. This review provides an overview on the current status of predictive biomarkers for histopathological response from NAC in LAGC, including clinicopathological variables, imaging and molecular testing. Furthermore, limitations and future perspectives are also discussed.

## INTRODUCTION

Gastric cancer is the fourth most common malignancy and the third most common cause of cancer morbidity and mortality according to GLOBOCAN2012[1]. Over the past decade, in-depth understanding of the biological mechanisms of gastric cancer has led to novel diagnostic, predictive, prognostic biomarkers and targeted therapies. Five-year overall survival of gastric cancer patients is still less than 25%, despite improved surgical and adjuvant approaches[2]. For locally advanced gastric cancer (LAGC) patients(stage II or higher, with no evidence of distant metastases, or locally advanced inoperable disease, as evaluated by CT, chest radiography, ultrasonography, or laparoscopy)[3], even comprehensive strategy including R0 resection with extended lymphadenectomy followed by adjuvant chemotherapy does not warrant long-term survival[4]; distant metastases and loco-regional recurrences still account for 40-51% of cases[5][6].

Tangible progress has been made in the area of therapeutics for LAGC. Neoadjuvant chemotherapy (NAC) is currently accepted worldwide as the initial treatment for LAGC, since its ability to facilitate curative surgery (R0 resection) as well as improve survival when combined with adjuvant chemotherapy was approved by two randomized phase III studies(MAGIC trial[3] and ACCORD-07 trial[7][8]). Those results prompted the National Comprehensive Cancer Network (NCCN) to adjust its treatment guidelines, recommending NAC as preferred option for LAGC (category 1 evidence) since the year 2008[9]. Although, a subsequent a phase III trial examined the value of purely NAC in LAGC patients with strict preoperative staging and standardized D2 resection failed to reach a significant survival benefit[10]. Indeed, two meta-analysis studies suggested that NAC could improve R0 resection rate and overall survival(OS), without affecting perioperative morbidity and mortality[11][12].

The underlying principles behind NAC is to increase R0 resection by shrinking /down-staging tumor and eliminating occult metastatic disease as early as possible[13][14]. Additionally, NAC can provide a valuable opportunity to test chemosensitivity *in vivo* and predict patient's respond to subsequent adjuvant chemotherapy[13]. Furthermore, NAC in clinical management of locally advanced operable tumor provides a useful platform for investigation and validation of potential predictive biomarkers, which is essentially important for tailoring individualized treatment[15].

**Figure 1:**
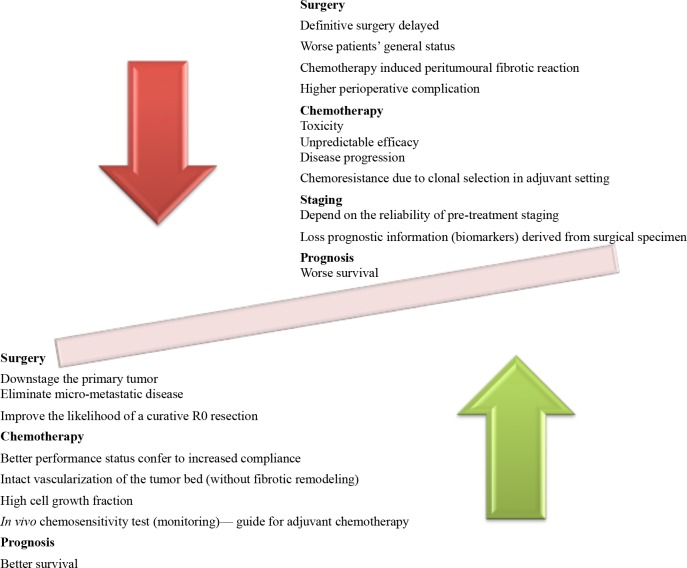
Summarized theoretic benefits and potential risks of neoadjuvant chemotherapy

However, the major clinical response rate after different NAC is only ranging from 20% to 45%[16]. Inter-individual differences in the response to NAC are currently observed among essentially all available NAC regimens. Such ‘unpredictable’ drug responses are particularly detrimental in the context of NAC. In other words, some patients undergo toxic, expensive, and fruitless chemotherapy in vain. Moreover, it is also possible that patients who are potentially curable by appropriate surgery would have progression of their disease while receiving NAC. Up to now, evidence supporting the idea that non-responder's prognosis by immediate surgical intervention is scarce. Nevertheless, the possibility of benefiting from upfront surgery in non-responders is low. Potential mechanisms might include, but not limited to, the following: chemotherapy-induced toxicity, chemotherapy-resistant tumor cell selection led to more invasive tumor cells and delayed surgical treatment[17][18]. In fact, patients who progress while on chemotherapy are unlikely to benefit from resection and can be spared radical surgery. The theoretic advantages and disadvantages of NAC are summarized in Figure 1. The long therapy developmental time period for NAC in gastric cancer over the last thirty years partially explains some of the skepticism about this treatment option[19]. Reliable predictive biomarkers for chemosensitivity which can be implemented before or shortly after chemotherapy are urgently needed in the NAC setting. Indeed, prediction of chemosensitivity with high accuracy is currently anticipated to further improve benefit from NAC[20][21].

The main goal of precision medicine is to predict patient's response to specific drugs. Identifying non-responders is crucial in avoiding/ or reducing potential harmful NAC. The premise of predicting chemotherapy response is that early prediction and detection of non-responsive tumors can prevent late and incurable disease. Therefore, a well-established strategy that allows predicting histopathological response after receiving NAC is crucial for implementation of NAC in LAGC patients. Unfortunately, standardized, readily accessible predictive assay remains scarce. The response rate to NAC is unmet and seems unpredictable. Although drug resistance in gastric cancer has been extensively explored in postoperative setting (reviewed in [22]). These kinds of results might be transferable to preoperative setting, but it is reasonable to speculate there are inherent difference between them, based on patients' tumor burden, tumor-host interaction, immune response, clinicopathological properties, and regimens they are administered. Their potential applicability may have to be readdressed in light of neoadjuvant setting. This review will focus on the current status of predictive biomarkers for chemotherapy in gastric cancer limited in neoadjuvant setting, discussing the direct evidence on predictive strategy for chemosensitivity from NAC.

We initially provide a brief overview on the existing histopathological response scoring system and then focus on the current status of biomarker research, including clinicopathological parameters, imaging studies, and molecular biomarkers that have shown promise and possible application in predicting tumor response to NAC in LAGC. Furthermore, future strategies for biomarker development in this field will also be discussed.

## LITERATURE SEARCH CRITERIA

PubMed and MEDLINE were searched for articles in English published before May 2015 using the terms “Gastric cancer”, “Neoadjuvant chemotherapy”, “preoperative chemotherapy”, “predictive biomarker”, “chemosensitivity”, “histopathological assessment”.

## HISTOPATHOLOGICAL ASSESSMENT

**Figure 2:**
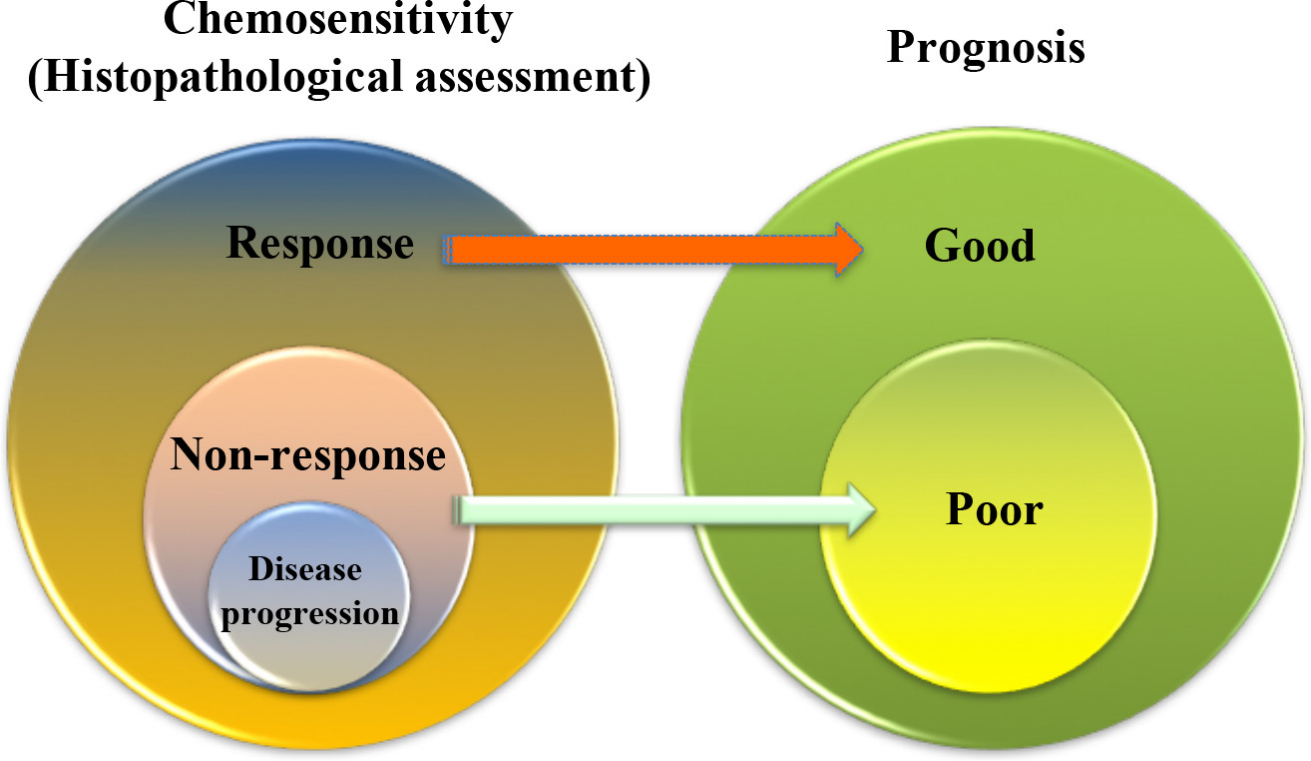
The correlation between histopathological response following neoadjuvant chemotherapy and long-term survival

Compared with adjuvant chemotherapy, NAC allows clinician to assess efficacy in relatively more objective and timely manner. Post-NAC tumor regression grade offers an alternative endpoint, which is the current gold standard for discriminating NAC responders from non-responders[15]. The best criterion and endpoint for the effectiveness of a specific anticancer therapy is survival and patient outcome. In line with this, it has been perceived that responders have a significantly better outcome since 1999[23]. Despite the fact that only a limited number of studies presented conflicting result[20][24][25], this conception was further confirmed by several subsequent studies[26][27][28][29]. Taken together, tumor response to NAC could be served as an independent prognostic factor for better prognosis (Figure 2). Most recently, based on data originating from two phase II trials of NAC, another study demonstrated that histopathological criteria (Japan criteria) is a better surrogate endpoint for overall survival than RECIST or Japanese Classification of Gastric Carcinoma (JCGC) criteria [30][31].

Post-operative histopathological response assessment (post hoc analysis) cannot be guide pre- and during the commencing course of NAC (endoscopy re-biopsy is difficult and inaccurate). Based on aforementioned studies, predictive biomarkers for tumor regression grade can provide additional information for individualizing treatment. Moreover, since the well-established correlation between NAC and histopathological regression, NAC is the crucial platform for developing novel predictive biomarkers and can subsequently be used for detailing adjuvant chemotherapy. Finally, taking advantage of NAC platform can facilitate the investigation on the correlation between patients' clinicopathological background and the effect of NAC. If some of the future additional large scale, prospective clinical studies further re-confirm that histopathological response is a reliable surrogate for overall survival, post-NAC histopathological assessment in gastric cancer could serve as the primary end point and lead to accelerating the regulatory approval of novel agents in this disease.

To date, the Becker[26], Ninomiya[32] and Mandard[33] scoring systems are the most frequently used for evaluating the histological response in gastric cancer treated with NAC (summarized in Table 1). All applied types of histopathology, to various degrees, are correlated with prognosis. These different criteria are considered to have the following two drawbacks: the lack of uniform standards between each other and the existence of observer-dependent bias(relevant observer-related)[13]. Mandard *et al*.[33] first established the histopathological regression for esophageal cancer post chemoradiotherapy. Subsequently, Becker *et al*. [26] modified Mandard's regression score to make it more adaptable to gastric cancer. Specifically, according to Becker's scoring system, histopathological responders are defined as patients with less than 10% residual tumor cells after NAC[28][34]. While another study proposed that only patients without residual tumor cells(complete tumor regression) could be considered to be histopathological responders[35]. On the contrary, patients with less than 50% residual tumor cells were classified by Shah *et al*.[36], Liu *et al*.[37] and Mansour *et al*.[38] as histopathological responders. Comparisons between studies and use of response criteria in routine practice are hampered by the lack of a universally accepted grading system[39].

**Table 1:** Histological response criteria following neoadjuvant chemotherapy in gastric cancers

Scoring system	Category	Criteria
Mandard	TRG 1	Absence of residual cancer and fibrosis extending through the layers of esophageal wall
	TRG 2	Presence of rare residual cancer cells
	TRG 3	Increase in number of residual cancer cells, but fibrosis still predominant
	TRG 4	Showing residual cancer out-growing fibrosis
	TRG 5	Absence of regressive changes
Japan(Ninomiya)	Grade 0	No change ± neither necrosis nor cellular or structural change can be seen throughout the lesion
	Grade 1a	Necrosis or disappearance of the tumour is present in less than 1/3 of the whole lesion
	Grade 1b	Necrosis or disappearance of the tumour is present in no more than 2/3 of the whole lesion
	Grade 2	Moderate change ± necrosis or disappearance of the tumour is present in more than 2/3 of the whole lesion, but viable tumour cells remain
	Grade 3	Marked change ± the whole lesion falls into necrosis and/or is replaced by fibrosis, with or without granulomatous changes. No viable tumour cells
Becker	1A	No residual tumour/tumour bed
	1B	<10% tumour cells
	2	10–50% residual tumour/tumour bed
	3	>50% no signs of neoplastic regression

Different scoring systems' reproducibility among various observers and their prognostic value were compared by Mirza *et al*[39]. Becker's scoring system was found to be the most reproducible for histological response assessment[39]. It is important to point out that even the Becker's criteria has high risk of inter-observer variability (κ-scores =0.51). Besides, only Mandard and Becker scores were found to be correlated with 5-year overall survival. Specifically, the 5-year survival rates were 100%(complete or nearly complete histopathological responders) and 35%(non-responders), respectively[39].

The homogenization of the various histopathological response assessment system will facilitate the comparison between different studies. Besides, since all aforementioned scoring systems only take residual tumor cells in the tumor bed into account, potentially neglecting the status of metastatic tumor cells regional to the lymph node. A previous study included involved and metastatic lymph nodes following neoadjuvant radiochemotherapy for esophageal cancer[40]. We suggested involved lymph nodes should also be evaluated in gastric cancer undergo NAC, which might present additional information. This hypothesis needs to be validated.

## CLINICOPATHOLOGICAL VARIABLES (SUMMARIZED IN TABLE 3)

Many clinicopathological variables have been correlated with histopathological response. Pre-treatment hemoglobin level and the presence of LN involvement were found to be related with histopathological response in a retrospective study of 119 gastric cancer patients treated with single-agent NAC(S1)[41]. Another study suggested TNM staging, histological type, tumor location, sex and age were correlated with histopathological response[27], however, the tumor regression related factors were not fully investigated in this study. Our previous retrospective study of 108 LAGC patients revealed that both tumor size and tumor differentiation were independent predictive marker for NAC responders, who were with better overall survival[28]. Interestingly, based on our updated database, a hypothesis-generating study found serum low-density lipoprotein measurement is useful in predicting chemosensitivity, higher low-density lipoprotein is statistically significant with histopathological response in LAGC patients undergoing NAC[42]. By analyzing 410 NAC treated LAGC, Lorenzen *et al* demonstrated that a predictive system, which is comprised of three pre-treatment clinicopathological variables (tumor localization, differentiation, and Lauren's classification), could predict chemosensitivity and prognosis [29].

Re-biopsies (second evaluation) taken during or after completion of NAC are proposed to be generally inaccurate (unreliable in the prediction of response)[26][43] and useful only in cases that are macroscopically highly suspicious, and can only be evaluated by post-operation histopathological examination. This technique may also be potentially quite risky, and certainly expensive, particularly if it is to be done on several occasions. Moreover, a negative result at histopathology does not prove that there is no tumor growth[15], since the biopsy might have been taken from an area of localized complete response. Moreover, staging laparoscopy has been shown to detect the presence of occult metastases approximately 8% to 26% of patients[44]. Pre and during the course of NAC, staging laparoscopy might detect not only pre-existing condition but also disease progression.

Up to our knowledge, prospective clinical trials focusing on investigation or validation of aforementioned or new predictive clinicopathological markers are urgently needed. These results generated from currently available studies are limited by their retrospective design, small sample size and various histopathological scoring criteria utilized. However, due to their ready availability, incorporation of these clinicopathological factors into approaching individualized treatment might be considered in clinical practice, if they are further revalidated by future prospective, large scale studies.

## IMAGE STUDIES (SUMMARIZED IN TABLE 3)

### Conventional anatomic imaging modalities

Morphologic based imaging modalities were considered to have disadvantage in assessing clinical response for gastric cancer patients. WHO criteria proposed that gastric cancer is not suitable for bi-dimensional evaluation[45]. On contrast, the one-dimensional based Response Evaluation Criteria in Solid Tumor (RECIST) criteria was considered to be applicable for gastric cancer[46]. Combination of endoscopy, computed tomography (CT) scans (with distention protocol) and endoscopy ultrasound (EUS) were implemented in assessing NAC treated patients during treatment or pre-operation. This comprehensive imaging modality was proved to be predictive of tumor regression at experienced centers[23][47][48][49]. Unfortunately, other studies revealed that morphologic imaging techniques, including CT and EUS, failed to accurately identify residual tumorous tissue within chemotherapy-treated areas due to occlusion by chemotherapy-induced fibrosis[50][51]. Be consistent with this, two other studies have also found that those conventional modalities were lacking of reliability for predicting response to NAC in esophageal cancer[52][43], might be due to chemotherapy induced edema and fibrosis. Besides, since the distension degree of the stomach is a determining factor in measuring gastric wall thickness, standardized distension protocol is essential for this procedure. Additionally, the formation of hyaline amorphous scar post chemo-induced tumor cell death and the connective tissue stromal component of the tumor making evaluation of viable tumor cell fraction in a residual mass difficult[53]. Moreover, chemotherapy or radiotherapy induced tumor size decline is considered to be a late event, which greatly limits the application of anatomic based imaging studies in predicting NAC response[53]. Another imaging study, which was based on a randomized phase II study, further re-confirmed these disadvantages of CT. The authors demonstrated that CT re-staging after NAC was inaccurate for gastric patients. In particular, the radiologic T-staging change after NAC could not be integrated into clinical decision-making process [54].

Nevertheless, currently, CT is the most widely utilized imaging modality for assessing response in gastric cancer patients treated with chemotherapy. The standardized protocol and parameters of CT for evaluating tumor response to treatment have been well established for these patients[50]. Preoperative clinical stage evaluation could provide prognostic information for gastric cancer patients[55]. Besides, it was proposed that clinical stage should be incorporated as a stratification factor in RCTs investigating preoperative therapy on patients with gastric cancer[55]. Moreover, low cost and readily accessibility enable CT to serve as the widespread utilized and standardized tools for identifying patients with disease progression during the course of NAC. Compared with measuring tumor diameter changes, volume changes calculated by CT demonstrated a higher correlation with early histopathological tumor regression to 2 weeks of NAC and a higher inter-observer consistency[56]. Moreover, the sensitivity and specificity of predicting histopathological response base on early volume change were 100% and 53%, respectively[56]. Be consistent with this, Lee *et al*. demonstrated that CT volumetry might be a reliable tool in the predicting tumor regression following NAC in patients with LAGC[57]. CT volumetry predicted pathologic response with surprisingly high accurate, being superior to standardized uptake values (SUV) taken from PET scans[57]. Their study suggested that patients, whose post-NAC volume reduction rate exceeds 35.6%, could be categorized as pathologic responders with 100% sensitivity[57].

EUS is thought to be an unreliable tool for response evaluation, as it is not able to distinguish between esophageal edema, fibrosis, and scarring from residual tumors[15]. In contrast, Guo *et al*. investigated the clinical feasibilities of EUS in predicting histopathological response after NAC. Patients with T and/or N down-staging(46%) had a relative more favorable pathological response to NAC than patients without T and/or N down-staging(54%)[58]. Be consistent with this, another study also showed that T and/or N down-staging by EUS was correlated with favorable overall survival and recurrence free survival. Taken together, T and/or N down-staging after NAC demonstrated by EUS may be served as a potential predictive marker for a favorable prognosis in patients with LAGC[59]. Recently, Ang *et al.* demonstrated that double enhance contrast ultrasound (DCUS) may represent an novel modality for more precisely predicting tumor regression[34].

These promising primary studies are still in their infancy, further validation are urgently needed. Nevertheless, they pave the way to utilizing readily available conventional imaging tools in predicting chemosensitivity from NAC. Implementation of strategies like ultrasound and CT could be especially beneficial for areas with limited resources.

MRI may also be useful in the prediction of pathological response following NAC in gastric cancer as it can effectively distinguish residual tumor from fibrosis or scar tissue. Recent studies have already demonstrated that MRI could accurately identify residual tumour in breast cancer patients underwent NAC[60]. Moreover, high-b-value diffusion-weighted MR imaging could detect relatively small effects (cell membranes permeability changes, cell swelling, and early cell lysis) in early stages of treatment[61]. Utilizing C26 colon cancer cell bearing mice treated with doxorubicin, Roth *et al* showed the potential application of this novel MR imaging for predicting treatment efficacy in an early stage[62]. Up to our knowledge, the usefulness of MRI in predicting pathological response after NAC has not been investigated in gastric cancer and warrants further study.

### Functional imaging (Metabolic Imaging studies)

Novel molecules based imaging technologies are being investigated to delineate the complexity, diversity and *in vivo* behavior of cancers while providing clinicians with new tools to tailor personalized treatment decisions[63]. Functional imaging may offer significant advantages in predicting histopathological response over conventional modality by identifying chemo induced alterations that precede a decline in tumor size[64]. Wahl *et al*. suggested using morphologic imaging techniques alone under WHO, RECIST, and RECIST 1.1 criteria has significant disadvantages and that both qualitative and quantitative approaches have been used to assess response from PET results[65]. Additionally, they proposed a new criteria, namely as PET Response Criteria in Solid Tumors (PERCIST), which could be served as an initial point for application in clinical trials and in structured quantitative clinical reporting[65].

The potential for PET scanning in investigating chemosensitivity is immense[66]. Fuorine-18 fluorodeoxy-glucose (^18^FDG), a well-established radiopharmaceutical for gauging exogenous glucose metabolism *in vivo*, is the most frequently used positron-emitting tracer for cancer imaging. Due to its incomplete intracellular degradation in cancer cells, ^18^FDG specifically accumulates in most malignant tumors, including gastric cancer[67]. The use of PET with ^18^FDG enables the visualization and quantification of responding areas in the tumor. Thus, the detection of ^18^FDG accumulation in residual tumor area by PET might predict histopathological response following NAC.

Indeed, PET can also monitor chemotherapeutic effects in gastric tumors by measuring changes in blood flow, metabolism, regional chemical composition, and absorption[68]. PET has already been utilized in predicting NAC efficacy by focusing on the molecular of characteristics cancer cells rather than anatomical properties alone.

Indeed, PET imaging was investigated on its ability to distinguish responding from nonresponding tumors in the early treatment stage of NAC for adenocarcinomas of the esophagogastric junction (AEG)[49]. In line with this study, ^18^FDG-PET was also proposed as a predictive marker in the MUNICON study, which proved the clinical feasibility of a PET-directed therapy optimization in AEG *via* early evaluation on tumor's metabolic change[69].

As early as 2003, Ott *et al*. prospectively evaluated the predictive value of ^18^FDG-PET for subsequent histopathological response in LAGC patients treated with NAC[48]. ^18^FDG-PET imaging accurately predicted histopathological response in 10 (77%) of 13 responders and 19 (86%) of 22 nonresponders through quantitative measurement of changes ^18^FDG uptake in relative early course of NAC(two weeks after treatment)[48]. Be consistent with this, Ott *et al*. also demonstrated that decrease of ^18^FDG uptake post-NAC(35% decrease as cut-off value) was correlated with tumor regression(less than 10% had residual tumors), which could predict the two year survival of the patients[70]. Similar results with different cutoff value was reported by another study. Shah *et al.* demonstrated that responders (less than 50% having residual tumors) were those with 45% decrease after 35 days post-NAC[36]. It has been proposed that defined cutoff values, standardized test methodology, and homogenized histopathological regression criteria should be established and validated before ^18^FDG-PET are clinically utilized[70].

Nevertheless, it is worthy to note that the utility of ^18^FDG-PET may be limited in evaluating gastric cancer of intestinal type and nonmucinous tumors[13][71]. Ott *et al.* showed that almost 40% of gastric cancers cannot be analyzed by ^18^FDG-PET due to insufficient contrast[13]. ^18^FDG-PET is not ideal tool for monitoring or predicting response in those ^18^FDG non-avid patients[70], whose overall survival were as poor as metabolic non-responders[13].

Improvement in accuracy might be achieved by implementing PET tracers other than ^18^FDG. Herrmann *et al.* showed that proliferative marker ^18^F-Fluorothymidine (^18^FLT) might be a potential feasible PET tracer[72]. ^18^FLT PET was consider to be superior than ^18^FDG PET in ^18^FDG non-avid patients[72]. The addition of ^18^FLT-PET to ^18^FDG-PET could improve early response prediction[70][73]. Moreover, Ott suggested ^18^FLT-PET might provide additional information on tumor proliferation status[73].

Due to unsatisfied sensitivity, a single PET scan has not been recommended for predicting post-NAC tumor regression in the upper gastrointestinal tract[74]. Serial PET scans, however, have limitations in terms of cost, availability and doses of radionuclide exposure.

Taken together, functional imaging studies are promising modality in individualizing treatment by characterizing tumor biology. However, there are still several issues that need to be resolved. Due to their technical complexity and high cost might be the biggest obstacle of their widespread implementation. Besides, novel tracers and potential specific molecular pathways should be developed and delineated for metabolic imaging.

## MOLECULAR TESTING(SUMMARIZED IN TABLE 4)

The promising molecular testing has become a critical component in the management of cancer patients[75][76]. In the era of precision medicine, predictive and prognostic biomarkers which can guiding clinical decision-making is in the center of current and future study. Great progresses have been achieved in clinically feasibility of molecular biomarkers. HER2 amplification status in breast and gastric cancer cells, BRAF mutation in colon cancers, KRAS and EGFR mutation in lung cancer have been widely implemented in clinical setting and improved patients' prognosis[77].

Potential genetic and epigenetic alterations which are involved in chemotherapeutic agents metabolism were thought be implicated in patients' responsiveness to chemotherapy[78]. Predictive biomarkers are of particularly significance for selecting NAC for individual patient, and the identification of molecular biomarkers which could predict histopathological response is crucial for the future use of NAC. Up to our knowledge, no clinically reliable predictive molecular biomarkers are currently available for personalizing LAGC patients' NAC treatment. The investigated molecular biomarkers that have proven to be of predictive value for histopathological response are discussed below.

### Genes involved in drug metabolism

In addition to taxane and anthracycline, the most frequently used drugs for NAC of LAGC are 5-Fu/ cisplatin-based. Accumulating studies have shown that 5-Fu or cisplatin metabolism involving pathways may influence patient outcomes following a 5-Fu/ cisplatin-based polychemotherapy.

DNA polymorphisms in the thymidylate synthase (TS) and 5,10-methylene-tetrahydrofolate reductase (MTHFR) genes, which are involved in the 5-FU pathway, were investigated by Ott *et al.* for their predictive value for histopathological response in LAGC treated with 5-FU based NAC[79]. Unfortunately, authors failed to find the statistically significant association between the TS or MTHFR genotypes and chemosensitivity[79].

Glutathione-S-transferase (GST) enzymes superfamily, which are important in metabolism (detoxification) of chemotherapy agents such as platin derivates[80]. GST-pi expression level of gastric cancer cell lines was proved to be correlated with chemosensitivity to cisplatin[81]. Ott *et al*. assessed GST polymorphisms as predictive markers for cisplatin-based NAC in LAGC[80]. Unfortunately, these was no significant correlation between the investigated GST polymorphisms or their combinations and chemosensitivity, showing GST polymorphisms could not distinguish response from nonresponse to NAC[80].

Excision repair cross-complementing 1 (ERCC1) is a key enzyme in the nucleotide excision repair (NER) pathway, and its expression was proposed as an predictive biomarker of the prognosis of advanced gastric cancer patients treated with platinum-based chemotherapy[82]. Napieralski *et al*. investigated whether seven therapy-related genes have the ability to predict the efficacy of 5-Fu/cisplatin-based NAC in LAGC patients[83]. The expressions of the 5-FU-related genes TS, Dihydropyrimidine dehydrogenase (DPD), Thymidine phosphorylase (TP) and of the cisplatin-related genes ERCC1, ERCC4, Ku autoantigen 80 (KU80), and Growth arrest and DNA-Damage-Inducible alpha (GADD45A) were tested by quantitative real-time PCR. Patients with higher TP and/or GADD45A values were exclusively found in nonresponding subgroup (*p* = 0.002). Significant correlation between DPD expression and histopathological response was also demonstrated, underlining the predictive value of DPD in 5-FU treated LAGC[83].

Damage DNA binding protein complex subunit 2 (DDB2) is the initial damage recognition molecule during nucleotide excision repair. Loss of DDB2 repair function contributes to cancer susceptibility and cellular sensitivity to DNA damage[23]. The potential correlation between the efficacy of DCS (docetaxel, cisplatin, and S-1) therapy and DDB2 and/or ERCC1 expression level of pretreated tumor tissues was examined[84]. The DDB2- and/or ERCC1-high phenotype was observed in 13 lesions (100%) of the nonresponders and in 7 lesions (25.9%) of the responders (*p* < 0.0001)[84]. Additionally, Fareed *et al*. revealed that ERCC1 nuclear expression correlated with histopathological non-response (*p* = 0.006) in gastro-oesophageal cancer patients who received platinum-based NAC[85].

### Other molecular markers

A statistically significant association of fractional allelic loss (FAL) with chemosensitivity was found, with a high FAL correlating with better therapy response in gastric cancer patients treated with cisplatin-based NAC[86]. Meanwhile, authors showed that *p53* mutation status was not an ideal predictive marker for NAC benefit[86].

Utilizing MethyLight technology, Napieralski *et al*. investigated the regional hypermethylation status of six tumor-related genes (promoter region), namely, MGMT, LOX, p16, E-cadherin, 14-3-3sigma and HPP1, for associations with therapy response and clinicopathologic features of 61 neoadjuvant-treated (5-FU/cisplatin-based) gastric cancer[78]. Their study demonstrated that a concordant methylation of more than three genes classified subgroups of gastric cancer with distinct biological and genetic characteristics. Methylation did not show a statistically significant correlation with response to cisplatin/5-fluorouracil-based NAC[78].

MicroRNA let-7i is a well established molecular invovled in chemo-resistance [87][88]. Liu *et al*. demonstrated that let-7i in pre-treatment tumor tissue might be a predictive marker for chemoensitivity in LAGC patients, wih low levels of let-7i correlating with poor histopathological response after NAC[37]. A double negative feedback loop between lin28 and let-7 has been intensively investigated. Manipulating the Lin28/let-7 pathway could provide novel therapeutic opportunities for treatment of cancer[89]. Consistent with these observation, our previous study revealed that Lin28 expression was significantly inverse associated with histopathological response, with higher expression of Lin28 was found in nonresponding patients[90].

Moreover, Death-associated protein-3 (DAP-3) was also found to be a useful predictive biomarker for predicting response to NAC in gastric cancer patients treated[91].

## LIMITATIONS OF CURRENT RESEARCH STRATEGY

Though marvelous progression has been made in predicting chemosensitivity of NAC in gastric cancer, some common limitations of recent studies still impeded further development. Results of these studies may be difficult to implement in clinical settings due to uncontrolled confounding variables in observational studies or rigid protocols in randomized trials. These points must be clearly addressed in future studies in order to facilitate discovery of predictive biomarkers of NAC response in LAGC. Specific limitations include but not limited:

Firstly, there is limited accuracy of current pre-treatment gastric cancer staging system. The disease progression after NAC for LAGC is not necessarily a late event of cancer progression when commencing NAC but more likely a pre-existing condition (for example staging laparoscopy can detect the presence of occult metastases) that was not appreciated before treatment.

Secondly, preliminary results, though promising, are based on relatively small sample sizes. There is an urgent need for large-scale, well-designed randomized controlled trials to aid our understanding of clinicopathological variables in predicting chemosensitivity to NAC. Since it is well accepted that the genetic background (Western vs Asia) influence gastric cancer patients' response to treatment, coming results generated from different populations should be interpreted with caution. To our knowledge, none of the aforementioned molecular biomarkers has been prospectively validated, and most studies, while well designed, have used small sample sizes. Consequently, measurement of molecular markers are still neither validated nor readily available in clinical settings.

Thirdly, substantial heterogeneity exits between the currently available studies and an optimal duration and regimen of neoadjuvant chemotherapy have not yet been established. Uniformity for the definition of LAGC is required to ensure the improved management of patients. To elaborate, there are no uniform inclusion criteria for tumor location(adenocarcinomas of esophagogastric junction and the lower third of the esophagus were included in some critical studies), tumor stage, surgical procedures, NAC regimen (including variable drug, dosage and course), and time point of response evaluation, even inhomogeneous histopathological assessment criteria. Lack of homogeneity makes comparison between studies difficult. Moreover, it is doubtful whether the results of studies with these faults can clearly determine whether or not one or another type of modality predicts histopathological response.

Fourthly, most of the molecular biomarkers are still in their infancy. Specific mechanisms should be characterized thoroughly and standardized readily accessible assays for these promising biomarkers should be developed. Clinical validation are urgently needed.

Finally, only one or two molecular biomarkers are investigated in most of the current studies. There is limited understanding of the molecular interactive functional networks[92].

## FUTURE PERSPECTIVES

Using predictive markers will hopefully eliminate unnecessary and potential harmful NAC. Identifying patient who is at high risk of loco-regional recurrence and distant metastasis, and targeting critical molecular or pathways will provide more opportunities to cure disease with lower toxicity[93]. On the other end of the spectrum, patients with molecular biomarkers suggesting low risk for local recurrence and/or distant metastasis, and those who are potentially curable but display markers indicating high possibility of resistance to NAC, could undergo upfront surgery[93].

The heterogeneity of gastric cancer is both the obstacle and opportunity for developing predictive and prognostic biomarkers. Different gastric cancer may need different types of treatment. As genomic and epigenomic studies evolve, further sub-classification of gastric cancer into new molecular entities is expected to facilitate treatment decision-making.

Promising and available break-through biomarkers which have been investigated in other cancers and may also be applicable in predicting response to NAC treatment of gastric cancer will also be addressed here. The integration of established clinicopathological indexes, imaging study and molecular testing with state-of-the-art molecular profiling will result in precise prediction of chemosensitivity from NAC in gastric cancer.

### Molecular subtype

Molecular classification of breast cancer have identified 3 distinct subclasses of breast cancer[94][95]. The molecular sub-types of breast cancer exhibits consistent prognostic significance and renders the development of therapeutic strategies[94][95].

Since the Lauren system of classifying gastric cancer was developed in 1965, the disease has been defined according to histologic features as either intestinal or diffuse. Can genetic data about gastric tumors provide additional information to inform therapy? Recently Shah *et al*. utilized GeneChip and bioinformatics to analyze cDNA expression of a gastric cancer specimen. Their study categorized gastric cancer into three different molecular types: proximal nondiffuse, diffuse, and distal nondiffuse gastric cancer. This sub-classification has implications for improving our understanding of unique molecular drivers of each gastric cancer type, aiding in the identification of novel predictive, therapeutic, prognostic biomarkers for each gastric cancer type[96]. Additionally, another group demonstrated that based on gene expression patterns, gastric cancers could be classified into 3 subtypes (proliferative, metabolic and mesenchymal)[97]. The subgroups exhibit distinguished molecular/genetic features and different response to therapy; this information might be utilized to individualize treatment approaches for gastric cancer patients[97]. Interestingly, the namely “metabolic” subtype are tend to be more sensitive to 5-Fu contained chemotherapy[97]. Meanwhile, the mesenchymal subtype might be more sensitive to inhibitors targeting the PI3K/AKT/mTOR pathway[97]. More recently, based on TCGA (The Cancer Genome Atlas) project, another study reported a comprehensive molecular characterization of 295 primary gastric cancer patients[98][98]. Their results sub-classify gastric cancer into four intrinsic molecular subtypes: EBV-infected tumors; MSI tumors; genomically stable tumors; and chromosomally unstable tumors[98]. More importantly, authors suggested that this sub-classification system might have the potential to guide targeted therapy for distinct subtypes of gastric cancer patients[98].

Gene-expression profiling based on high throughput sequence can be used to classify gastric cancers into different subtypes, which have differences in molecular and genetic features. These differences may also correlate with response to specific treatments. Albeit, whether this kind of promising molecular subclassification of gastric could implicit in predicting chemosensitivity from NAC in LAGC is still unknown. It is rationale to propose that comprehensive molecular characterization of gastric cancer patients could be served as a promising approach and potentially lead to more-effective, personalized therapy.

### Next generation sequencing

Genomic, epigenomic, transcriptomic sequencing data generated from Next Generation Sequencing (NGS) might be implemented to classify molecular which are involved in patients' responses to NAC. Besides, further reduction in costs and improvements in technology will make full classification of cancer genomes clinically feasible[99][100]. With this kind of trend, this revolutionary approach will be available in routine clinical use and change the way we treat patients in the not too distant future. Optimizing treatment pre and/or during NAC for individual patients can be realized by deeper understanding of the genomics of gastric cancer. Incorporation of this platform into clinical studies and eventually standards of routine clinical care should aid in tailoring individualized strategy.

Moreover, other high throughput technologies, including pharmacogenomic, proteomic and metabolomic, might also shed lights on the future of cancer care. These data has high possibility to document the uniqueness of tumors in regard to treatment response[101]. Using ever faster, ever-cheaper sequencing methods and heavy-duty bioinformatics will enlarge the catalogue of molecular marker changes associated with tumor and treatment response.

Evaluation of these genomic and epigenomic alterations only offers a snapshot of the cancer at a specific time point. As the tumor evolves, more alteration will occur and the heterogeneity of the tumor will increase[102]. Understanding the genomic and epigenetic alterations of each tumor in a dynamic way might have the promise of solving this challenge.

### Circulating tumor cells and circulating tumor DNA

The potential of circulating tumor cells (CTCs) in the early and non-invasive monitoring of response to therapy has become clear in the past few years[103][104]. The latest approaches to CTC capture and molecular profiling, including next-generation sequencing, mutation analysis, proteomic profiling, single-cell analysis and mutational heterogeneity analysis might further enhance its use.

Most recently, Dawson *et al*. showed that compared with CA 15-3 or circulating tumor cells, circulating tumor DNA(ctDNA) levels change of serially collected plasma specimens in metastatic breast cancer patients demonstrated a more closely association with tumor burden changes. Among the measures utilized, ctDNA was proved to be the earliest to predict tumor response to treatment in metastatic breast cancer patients [105]. This proof-of-concept analysis showed that circulating tumor DNA is an informative, inherently specific, and highly sensitive biomarker of metastatic breast cancer. These findings allow physicians to continually adjust treatment strategies as the tumor progresses. Quickly identification of the detailed characteristics of the tumor, which is helpful to select the effective treatment to target the evolved tumor cells[105].

Although this is only a preliminary report of a small group of patients and requires confirmation by others, this study provides a provocative view of the future of cancer monitoring.

## CONCLUSION

In patients with LAGC, precisely Predictive biomarkers can identify tumors that are more likely to respond to specific targeted treatments, and they allow us to avoid ineffective options. The inability to select similarly for or against chemotherapy use, coupled with the toxic effects, costs, and inconvenience of chemotherapy, has been a growing source of concern.

predicting the response to NAC remains lacking reliable tools. The unmet histopathological response rate from NAC highlights the need to explore integrated clinicopathological parameters, imaging studies and molecular markers (Tables 2–4) that might identify patients with a high probability of response in order to avoid unnecessary and ineffective treatment in patients unlikely to respond. In any case, the ultimate decision to administer NAC would require a biomarker or combination of biomarkers with high specificity and a high positive predictive value.

**Table 2:** Investigated Clinicopathological variables for neoadjuvant chemotherapy in patients with LAGC

Clinicopathological variable	Brief summarization	Reference
Hemoglobin level	Hemoglobin level was related to the response	[41]
Lymph node metastasis	The presence of lymph node metastasis was correlated with NAC Chemosensitivity	[41]
Tumor size	Tumor size was independent predictors of tumor regression	[28]
Tumor localization	Tumor localization in the middle third of the stomach was related to the response	[29]
Differentiation	Well tumor differentiation were related to better histopathological response	[29]
Laure's classification	Intestinal tumor type according to Lauren's classification accurately predicts histopathological response and prognosis in neoadjuvant treated LAGC	[29]
Serum low-density lipoprotein	Higher low-density lipoprotein is statistically significant with histopathological response in LAGC patients undergoing NAC	[42]
Endoscopy(second evaluation)	Re-biopsies taken after NAC do not help in determining the response, since the biopsy might have been taken from an area of localized complete response	[15]
Laparoscopy(re-staging)	Staging laparoscopy might detect not only pre-existing condition but also disease progression during NAC	[43]

**Table 3:** Investigated image studies for neoadjuvant chemotherapy in patients with LAGC

Modality	Brief summarization	Reference
Endoscopy ultrasound (EUS) Computed tomography (CT)	The reliability of EUS and CT in predicting NAC response is still controversial. CT volumetry reduction, T and/or N down-staging by EUS and double enhance contrast ultrasound (DCUS) might be promising modalities.	[23][47][48][49] [50][51][52][43] [34][56][57][58][59]
Magnetic Resonance (MR)	Usefulness of MRI in predicting pathological response after NAC has not been investigated in LAGC, High-b-value diffusion-weighted MR imaging might be promising tool.	[61][62]
Positron emission tomography (PET)	18FDG-PET allow early differentiation of responding and nonresponding tumors during NAC, despite are non-avid 18FDG non-avid patients(intestinal type and nonmucinous tumors) are not suitable for response monitoring using the PET tracer 18FDG. Adding 18FLT-PET to 18FDG-PET might improve early prediction of response to NAC.	[36][48][70] [13][71] [70]

**Table 4:** Investigated molecular markers for neoadjuvant chemotherapy in patients with LAGC

Predictive molecular markers	Brief summarization	Reference
Glutathione-S-transferase (GST)	Overexpression of GST showed a significantly better sensitivity to cisplatin-based NAC	[81]
Dihydropyrimidine dehydrogenase (DPD)	Significant correlation between DPD expression and histopathological response	[83]
TP and/or GADD45A	High expression values of TP and/or GADD45A were exclusively found in nonresponding patients	[83]
Damage DNA binding protein complex subunit 2 (DDB2)	DDB2- and/or ERCC1-high phenotype was significantly correlated with nonresponding patients	[84]
Excision repair cross-complementing 1 (ERCC1)	ERCC1 nuclear expression correlated with lack of histopathological response	[85]
Fractional allelic loss (FAL)	High FAL value) was shown to define a subset of gastric cancer patients who were more likely to benefit from cisplatin-based NAC	[86]
Let-7i	Low levels of let-7i were significantly correlated with poor histopathological response after NAC	[37]
Lin28	Higher expression of Lin28 was found in nonresponding patients after NAC	[90]
Death-associated protein-3 (DAP-3)	DAP-3 correlated with NAC effectiveness and prognosis of gastric cancer patients following	[91]

**Figure 3:**
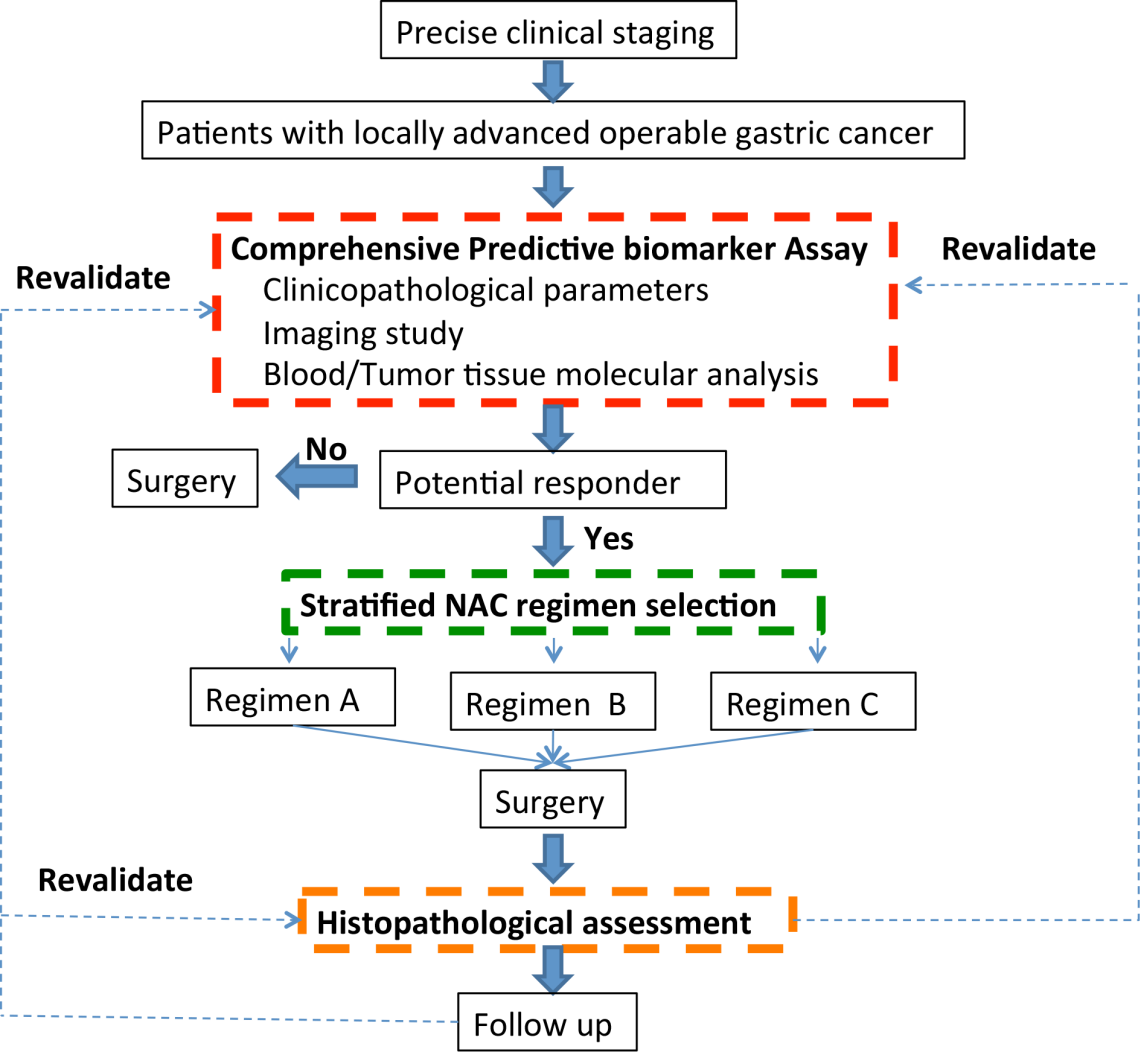
A proposed dynamic model for predicting response from neoadjuvant chemotherapy

The immediate future will be focused on integrating these new strategies in light of characterizing of the potential molecular mechanisms of gastric cancer and identifying optimal treatment choices for a given individual with the ongoing quest of identifying validated predictive biomarkers. Further investigation and validation of the existing promising candidate biomarkers are urgent guaranteed. Meanwhile, there is also an urgent need to continue exploration on novel molecular markers. We propose a combined and integrated approach, which is consisted of clinical, imaging, and molecular biomarkers (Figure 3). Large, well-designed prospective trials are desirable to pinpoint key potential candidate biomarkers to facilitate clinician's strategy based on a patient's unique profile. These predictive biomarkers, like signposts, are needed to guide a clinician's treatment decision for LAGC patients. These decisions should not feel like gambles but should be reasoned choices, grounded in personalized insight and an understanding of existing scientific knowledge. The current ‘blunderbuss’ approach will be replaced by precise one.
